# Obituary

**Published:** 1864-04

**Authors:** 


The following obituary of Dr. York has been kindly furnished us by his intimate friend, B. F. Swafford, M. D., of New Goshen, Ind.

Dr. Shubal York, died by the hand of an assassin, at Charleston, Illinois, March 28th, 1864.

Dr. York was born in Randolph County, North Carolina, about the year 1816. His parents, though respectable, were in moderate circumstances; hence all the education they were able to give their son was such as the common schools of the country afforded. Young York emigrated with his parents to Illinois, in the year 1834; taught school for a while, and then entered the office of Drs. Steele & Willard as a student of medicine. After a course of office instruction he began practice in 1840, without taking his degree; he, however, graduated at the Cleveland Medical College, some years later, with high honors.

Both in his profession and in the sciences which go to make up a sound education, Dr. York was a man of extensive learning; in the former he was decidedly a progressive man, always fully up to times, and gathering information from every available source. On the breaking out of the rebellion Dr. York took decided grounds in favor of his country, left his lucrative practice and quiet home to accept a position as Surgeon of the 54th Ill. Vol. Infty., which position he held with honor to himself and advantage to the service until his death. Dr. York was plain in his dress, temperate in his habits, unaffected in his manners, in conversation witty and vivacious, as an orator, eloquent and pungent, as a debater, adroit and logical.

But it was in private life where he manifested those qualities which won him so many friends; whether we consider him as a Christian, a citizen, or a humanitarian, we find him discharging all duties with a conscientiousness rarely met with among men. Though he now sleeps peacefully in the last resting place of man, his memory will ever be cherished until those who knew him sleep with him. We can only say, Devoted Christian, Generous Friend, Courteous Gentleman, farewell.

---

Death of Dr. O. L. Leonard.—At the meeting of the Chicago Medical Society, on Friday, April 1st, the following resolutions were adopted:

Whereas, in the order of Divine Providence, our friend and colleague, Dr. O. L. Leonard, has been called from our midst,

*Resolved*, That, while deploring his death, as the loss of a Christian gentleman, and a kind and faithful fellow laborer, we bow with humility before the will of Him who is alike infinite in wisdom and beneficence.

*Resolved*, That we recognized in our departed Brother those honorable and noble qualities of the mind and heart that endeared him alike to his family, his patients, and his confreres.

*Resolved*, That we hereby tender our heartfelt sympathies to the family of our deceased associate.

*Resolved*, That the Secretary of this Society be directed to present a copy of these resolutions to the relatives of the Deceased, and to request their publication in the Medical Journals and the newspapers of this city.

E. Marguerat, M. D., *Sec'y.*

Death of Dr. Franklin Bache.—Our readers will hear with regret of the demise of this distinguished physician and chemist, which occurred at his residence in Philadelphia on Saturday afternoon, March 19th. Dr. Bache had been ill but about a week, and had shown no symptoms of declining health until the attack which terminated his long and useful life. He delivered his full course on chemistry in Jefferson Medical College during the past winter, and was even present at the party given to the graduating class on the evening of the 10th of March. By his death the medical profession loses one of its brightest ornaments, a man pre-eminently distinguished for his sterling integrity and uncompromising adherence to truth—the first requisites in a man of science. Dr. Bache was born in Philadelphia, October 25, 1792; he graduated as bachelor of arts in 1810 at the University of Pennsylvania, and four years afterwards took his degree in the medical department of the same Institution.

He served several years in the army as surgeon, and afterwards commenced practice in Philadelphia, occupying several medical positions, and lecturing from 1826 to 1832 in the Franklin Institute of Philadelphia. In 1831 he succeeded Dr. Wood as Professor of Chemistry in the Philadelphia College of Pharmacy, Dr. Wood being transferred to the chair of Materia Medica. The intimacy formed betwe n these two eminent men led them to co-operate in the production of a much needed work for which they possessed remarkable qualifications, and in 1833 the United States' Dispensatory appeared. Its success, and the manner in which it has informed and educated the physicians and pharmaceutists of the United States, are matters of history. The U. S. Dispensatory, after numerous revisions, continues now the indispensable handbook of every pharmaceutist and physician in this country, and is widely diffused in Europe and wherever the English language is read. In 1841 Dr. Bache wes elected Professor of Chemistry in the Jefferson Medical College, a position he occupied till the time of his death. He also enjoyed many evidences of the esteem of his fellow-citizens and the profession; was an ex-President of the American Philosophical Society; Vice President of the College of Physicians of Philadelphia, and President of the Deaf and Dumb Asylum.

Dr. Bache was the eldest son of the eldest grandchild of the great Dr. Franklin, and inherited some of the traits of his illustrious ancestor, which will place his name in a high position among American Philosophers.—*Am. Drug. Circular.*

